# Supplementary material for: Contents and Correlations of Nε-(carboxymethyl)lysine, Nε-(carboxyethyl)lysine, Acrylamide and Nutrients in Plant-Based Meat Analogs
Source: Foods. 2023 May 12;12(10):1967. doi: 10.3390/foods12101967 (PMC10217484; doi:10.3390/foods12101967)
Supplement: Supplementary file 1 [file foods-12-01967-s001.zip › foods-2374894-supplementary.pdf]

Table S1. Retention times and MS/MS parameters of CML, CEL, acrylamide, and their isotope-labeled internal standards by multiple reaction monitoring (MRM).

compounds	retention time (min)	precursor ion (m/z)	product ion (m/z)	collision energy (ev)	cone voltage (v)
CML	2.82	205	84*	16	4
			130	10	4
CEL	2.99	219	84*	20	2
			130	12	2
acrylamide	1.68	72	55*	8	20
			44	10	20
CML-d_4	2.81	209	88*	18	2
CEL-d_4	2.98	223	88*	18	2
acrylamide-d_3	1.66	75	58*	10	22

Product ion marked with * was used for quantification, the other product ion was used for confirmation.

Table S2. Individual amino acids contents of plant-based meat analogs (g/100 g).

	Asp	Thr	Ser	Glu	Gly	Ala	Cys	Val
A	4.31 ± 0.12^{c}	1.49 ± 0.04^{de}	1.92 ± 0.05^{d}	8.94 ± 0.26^{de}	1.53 ± 0.03^{f}	2.33 ± 0.04^{cd}	0.73 ± 0.02^{efg}	1.69 ± 0.04^{de}
B	4.83 ± 0.12^{b}	1.63 ± 0.05^{cd}	2.19 ± 0.09^{bc}	7.73 ± 0.22^{gh}	1.69 ± 0.02^{e}	1.93 ± 0.05^{e}	0.75 ± 0.06^{ef}	2.14 ± 0.06^{ab}
C	4.49 ± 0.04^{bc}	1.63 ± 0^{cd}	2.01 ± 0.03^{cd}	9.84 ± 0.08^{bc}	1.64 ± 0.02^{e}	1.75 ± 0.02^{gh}	1.18 ± 0.02^{ab}	1.90 ± 0.04^{cd}
D	4.65 ± 0.09^{bc}	1.63 ± 0.03^{c}	2.07 ± 0.05^{bcd}	7.41 ± 0.09^{h}	1.67 ± 0.02^{e}	1.92 ± 0.04^{e}	0.75 ± 0.12^{efg}	2.11 ± 0.08^{abc}
E	4.72 ± 0.08^{b}	1.71 ± 0.06^{bc}	2.19 ± 0.04^{bc}	9.30 ± 0.11^{cd}	1.73 ± 0.01^{e}	1.90 ± 0^{ef}	0.99 ± 0.06^{bcd}	1.97 ± 0.05^{bc}
F	4.61 ± 0.14^{bc}	1.70 ± 0.05^{bc}	2.21 ± 0.02^{b}	8.38 ± 0.24^{ef}	1.84 ± 0.04^{d}	2.24 ± 0.06^{d}	1.10 ± 0.03^{bc}	2.17 ± 0.12^{ab}
G	3.54 ± 0.11^{d}	1.37 ± 0.02^{ef}	1.92 ± 0.02^{d}	8.33 ± 0.19^{efg}	1.43 ± 0.02^{gh}	1.61 ± 0.05^{hi}	0.83 ± 0.09^{de}	1.73 ± 0.14^{de}
H	3.83 ± 0.10^{d}	1.44 ± 0.04^{e}	1.94 ± 0.06^{d}	8.19 ± 0.20^{fg}	1.50 ± 0.01^{fg}	1.53 ± 0.03^{ij}	0.77 ± 0.06^{ef}	1.53 ± 0.02^{ef}
I	2.10 ± 0.03^{f}	0.87 ± 0.01^{h}	1.20 ± 0.01^{f}	5.87 ± 0.11^{i}	0.85 ± 0.01^{j}	1.03 ± 0.01^{k}	0.91 ± 0.05^{cde}	1.29 ± 0.03^{gh}
J	5.77 ± 0.32^{a}	2.11 ± 0.12^{a}	2.47 ± 0.17^{a}	9.78 ± 0.49^{c}	2.18 ± 0.07^{b}	2.39 ± 0.13^{c}	1.35 ± 0.12^{a}	2.26 ± 0.13^{a}
K	4.62 ± 0.09^{bc}	1.78 ± 0.03^{b}	2.51 ± 0.09^{a}	11.17 ± 0.34^{a}	2.38 ± 0.03^{a}	2.95 ± 0.04^{a}	1.36 ± 0.13^{a}	2.11 ± 0.04^{abc}
L	3.77 ± 0.08^{d}	1.24 ± 0.04^{fg}	1.67 ± 0.04^{e}	6.17 ± 0.11^{i}	1.36 ± 0.03^{h}	1.82 ± 0.04e^{fg}	0.53 ± 0.04^{g}	1.39 ± 0.05^{fg}
M	3.02 ± 0.07^{e}	1.15 ± 0.04^{g}	1.61 ± 0.04^{e}	7.79 ± 0.15^{fgh}	1.55 ± 0.03^{f}	1.77 ± 0.03^{fg}	0.70 ± 0.06^{efg}	1.33 ± 0.05^{fg}
N	4.55 ± 0.03^{bc}	1.73 ± 0.01^{bc}	2.44 ± 0.02^{a}	10.48 ± 0.08^{b}	1.99 ± 0.01^{c}	2.76 ± 0.03^{b}	1.37 ± 0.04^{a}	2.12 ± 0.05^{ab}
O	2.39 ± 0.01^{f}	0.91 ± 0.01^{h}	1.28 ± 0.02^{f}	5.80 ± 0.04^{i}	1.05 ± 0.01^{i}	1.46 ± 0.01^{j}	0.6 ± 0.02^{fg}	1.11 ± 0.02^{h}
	**Met**	**Ile**	**Leu**	**Tyr**	**Phe**	**Lys**	**His**	**Arg**
A	0.04 ± 0^{e}	1.66 ± 0.09^{d}	3.09 ± 0.1^{fg}	1.11 ± 0.03^{fg}	1.89 ± 0.04^{e}	2.76 ± 0.06^{cd}	0.94 ± 0.01^{fg}	2.75 ± 0.05^{e}
B	0.06 ± 0^{d}	1.91 ± 0.04^{b}	3.46 ± 0.07^{cd}	1.50 ± 0.05^{g}	2.34 ± 0.05^{b}	3.28 ± 0.1^{b}	1.06 ± 0.01^{d}	3.37 ± 0.07^{b}
C	0.03 ± 0^{fg}	1.72 ± 0.05^{cd}	2.97 ± 0.06^{gh}	1.25 ± 0.03^{de}	2.03 ± 0.05^{d}	2.92 ± 0.04^{c}	0.95 ± 0.01f^{g}	2.52 ± 0.05^{fg}
D	0.07 ± 0^{d}	1.90 ± 0.02^{b}	3.34 ± 0.05^{de}	1.45 ± 0.03^{bc}	2.23 ± 0.03^{bc}	3.26 ± 0.06^{b}	1.01 ± 0.01^{e}	3.23 ± 0.02^{bc}
E	0.04 ± 0e^{f}	1.88 ± 0.0 ^{bc}	3.25 ± 0.11^{ef}	1.37 ± 0.11^{cd}	2.20 ± 0.07^{c}	3.15 ± 0.05^{b}	1.12 ± 0.01^{c}	3.03 ± 0^{d}
F	0.04 ± 0^{e}	1.90 ± 0.10^{b}	3.50 ± 0.08^{cd}	1.57 ± 0.03^{ab}	2.31 ± 0.05^{bc}	3.14 ± 0.05^{b}	1.13 ± 0.02^{b}	3.37 ± 0.07^{b}
G	0.48 ± 0.01^{b}	1.56 ± 0.08^{de}	2.90 ± 0.07^{gh}	1.15 ± 0.02^{efg}	1.96 ± 0.03^{de}	2.13 ± 0.05^{f}	0.90 ± 0.01^{g}	2.44 ± 0.03^{h}
H	0.02 ± 0^{gh}	1.44 ± 0.01^{ef}	2.82 ± 0.04^{h}	1.22 ± 0.05^{ef}	1.89 ± 0.03^{e}	2.62 ± 0.03^{de}	0.96 ± 0^{f}	2.65 ± 0.03^{ef}
I	0.37 ± 0.01^{c}	0.88 ± 0.01^{h}	1.68 ± 0.02^{k}	0.59 ± 0.02^{i}	1.16 ± 0.03^{h}	1.27 ± 0.02^{g}	0.50 ± 0.01^{j}	1.32 ± 0.02^{j}
J	0.02 ± 0gh	2.12 ± 0.08^{a}	3.97 ± 0.12^{a}	1.64 ± 0.09^{a}	2.71 ± 0.07^{a}	3.87 ± 0.14^{a}	1.35 ± 0.02^{a}	3.69 ± 0.12^{a}
K	0.02 ± 0^{gh}	1.98 ± 0.06^{ab}	3.67 ± 0.07^{bc}	1.61 ± 0.05^{a}	2.62 ± 0.03^{a}	3.15 ± 0.07^{b}	1.19 ± 0.02^{b}	3.21 ± 0.05^{c}
L	0.01 ± 0^{h}	1.40 ± 0.02^{ef}	2.61 ± 0.04^{i}	1.07 ± 0.02^{abc}	1.75 ± 0.04^{f}	2.53 ± 0.04^{e}	0.83 ± 0.01^{h}	2.62 ± 0.04^{ef}
M	0.02 ± 0^{gh}	1.34 ± 0.02^{f}	2.49 ± 0.04^{i}	1.02 ± 0.02^{g}	1.69 ± 0.02^{f}	2.02 ± 0.04^{f}	0.79 ± 0.03^{h}	2.05 ± 0.04^{h}
N	0.78 ± 0^{a}	2.09 ± 0.06^{a}	3.73 ± 0.05^{b}	1.63 ± 0.02^{a}	2.60 ± 0.02^{a}	2.63 ± 0.03^{de}	1.15 ± 0.01^{bc}	3.04 ± 0.03^{d}
O	0.36 ± 0.01^{c}	1.11 ± 0.01^{g}	1.96 ± 0.01^{j}	0.76 ± 0.01^{h}	1.34 ± 0.01^{g}	1.34 ± 0^{g}	0.60 ± 0.01^{i}	1.56 ± 0.01^{i}

Values are expressed as mean ± standard deviation (SD) (n = 3). Values in the same row marked by different letters indicate statistically different at 0.05 level.

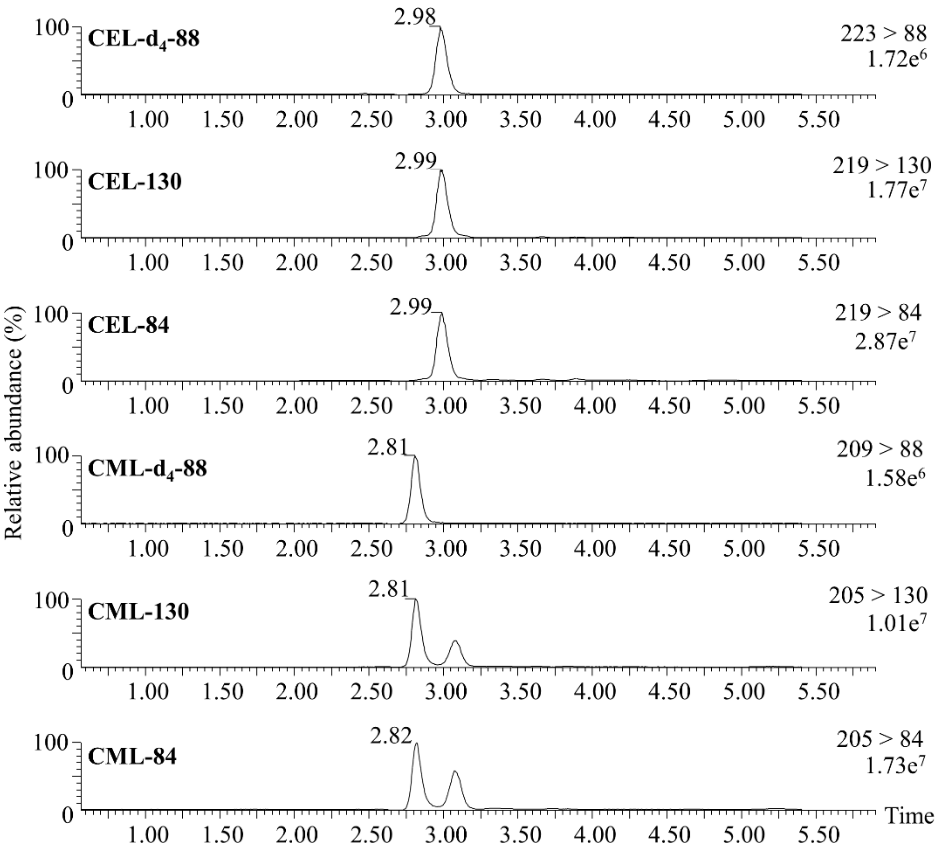

Figure S1. UHPLC-MS/MS chromatograms of CML and CEL, and their respective internal standards in a plant-based meat analog obtained by multiple reaction monitoring in positive ionization mode.

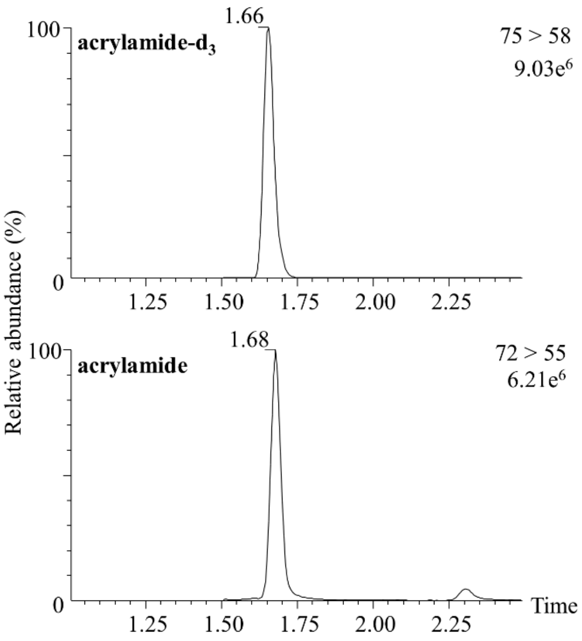

Figure S2. UHPLC-MS/MS chromatograms of acrylamide and its internal standard in a plant-based meat analogs obtained by multiple reaction monitoring in positive ionization mode.
